# Incidence and Effect of Intrathecal Fentanyl Use in Spinal Anesthesia for Cesarean Deliveries in the Community Setting: A Single-Center Observational Retrospective Study

**DOI:** 10.31486/toj.20.0147

**Published:** 2021

**Authors:** Autumn Brewer, Sarah Joseph, Kendall Hammonds, Michael P. Hofkamp

**Affiliations:** ^1^ Texas A&M University Health Science Center College of Medicine, Temple, TX; ^2^ Office of Biostatistics, Baylor Scott & White Research Institute, Temple, TX; ^3^ Department of Anesthesiology, Baylor Scott & White Medical Center – Temple, Temple, TX

**Keywords:** *Analgesia*, *anesthesia–conduction*, *anesthesia–obstetrical*, *cesarean section*

## Abstract

**Background:** The addition of intrathecal fentanyl to spinal anesthesia for cesarean delivery has been shown to be beneficial, but its rate of utilization in the community setting is unknown. The primary aim of our study was to determine the rate of intrathecal fentanyl use for cesarean deliveries with spinal anesthesia in a community hospital, and our secondary aim was to determine its effect on anesthetic outcomes.

**Methods:** Patients who underwent cesarean delivery from June 1, 2017 to November 30, 2019 with spinal anesthesia as the initial anesthetic technique were included.

**Results:** Seven hundred sixty-one cesarean deliveries met inclusion criteria, and 161 (21.2%) patients received intrathecal fentanyl in their spinal anesthetic for cesarean delivery. A multivariate model that controlled for patient weight and time from spinal placement to procedure end showed that patients who received intrathecal fentanyl were less likely to have conversion to general anesthesia or administration of systemic anesthetic adjuncts compared to patients who did not receive intrathecal fentanyl (odds ratio 2.889, 95% CI 1.552-5.903; *P*=0.0017).

**Conclusion:** Only 1 in 5 patients received intrathecal fentanyl for cesarean deliveries performed under spinal anesthesia in a community hospital despite known benefits. Patients who did not receive intrathecal fentanyl had increased odds of conversion to general anesthesia or administration of systemic anesthetic adjunct administration, a finding consistent with previous studies. The addition of intrathecal fentanyl to spinal anesthesia for cesarean delivery should be strongly considered in the community setting.

## INTRODUCTION

Anesthesia for cesarean delivery is commonly performed with spinal anesthesia that consists of a local anesthetic such as bupivacaine, a short-acting opioid such as fentanyl, and a long-acting opioid such as morphine.^1^ Intrathecal fentanyl has been shown to be beneficial for intraoperative pain associated with cesarean delivery,^2^ while intrathecal morphine has an important effect on postoperative pain.^3^

Conversion to general anesthesia signifies failure of spinal anesthesia for cesarean delivery, while the administration of systemic anesthetic adjunct medication such as intravenous fentanyl or inhaled nitrous oxide is suggestive of inadequate analgesia. Clevenger and colleagues found that 13.9% of patients who had spinal anesthesia as their primary anesthetic technique for cesarean delivery required the use of systemic anesthetic adjuncts.^4^

The prevalence of intrathecal fentanyl administration in spinal anesthesia for cesarean delivery in the community setting is unclear. The primary aim of our study was to determine the rate of intrathecal fentanyl use in spinal anesthesia for cesarean delivery in a community setting. The secondary aim was to determine if a difference in either conversion to general anesthesia or administration of systemic anesthetic adjunct administration was found between patients who did and did not receive intrathecal fentanyl for cesarean delivery.

## METHODS

After approval by the Baylor Scott & White Research Institute Institutional Research Board, the electronic medical record system (Epic Systems Inc) was used to search for cesarean deliveries performed at Baylor Scott & White Medical Center – College Station from June 1, 2017 to November 30, 2019. Patients were included if they received spinal anesthesia as the initial anesthetic technique for cesarean delivery and were excluded if they received labor epidural analgesia. Study data were collected and managed using REDCap electronic data capture tools hosted at Baylor Scott & White Research Institute. Demographic data including age, height, weight, body mass index, gravidity, parity, history of previous cesarean delivery, and gestational age were collected. The principal investigator examined each operative note and categorized the obstetric indication of cesarean deliveries into elective, urgent, and fetal heart rate abnormalities based on whether the cesarean delivery was scheduled, unscheduled, or had fetal heart rate abnormalities documented. The attending anesthesiologist of record was recorded by assigning the letter A to the first attending anesthesiologist who appeared in the study and the letter B to the second, with the process repeated through the letter J. Date and time of spinal placement for cesarean delivery and the end of the procedure were collected. Additional data collected were the neuraxial anesthetic kit used, whether the operator performing the neuraxial anesthetic was a physician anesthesiologist or certified registered nurse anesthetist, dose of hyperbaric bupivacaine 0.75%, dose of intrathecal preservative-free morphine, dose of intrathecal fentanyl, whether the patient required conversion to general anesthesia, and whether the patient received anesthetic adjuncts during the cesarean delivery (intravenous fentanyl, morphine, ketamine, propofol, midazolam, inhaled nitrous oxide, and sevoflurane).

Statistical analysis was performed using SAS statistical software, version 9.4 (SAS Institute Inc). Descriptive statistics are used to describe characteristics of the sample. Frequencies and percentages are used to describe categorical variables, and medians with interquartile ranges are used to describe continuous variables. Chi-square test, Fisher exact test, or Monte Carlo estimated Fisher test was used for categorical variables, and Wilcoxon rank sum test was used to test for continuous variables to examine associations in bivariate comparisons. Multivariate logistic regression was used to examine the effect of intrathecal fentanyl use on either conversion to general anesthesia or administration of systemic anesthetic adjunct administration while controlling for selected clinically and statistically significant variables. Variables with a bivariate *P* value <0.10 were considered for inclusion in the final model. All potential covariates were then included in a logistic regression and removed one at a time until only clinically significant or those with a *P* value <0.10 variables remained. Statistical significance was determined at a level of 0.05.

## RESULTS

Seven hundred sixty-one cesarean deliveries met inclusion criteria for the study. Although some anesthesiologists and certified registered nurse anesthetists used a combined spinal epidural neuraxial kit to obtain spinal anesthesia, no epidural catheters were inserted into the epidural space. Six hundred patients did not receive intrathecal fentanyl, and 161 patients did: 4, 153, and 3 patients received 5 μg, 10 μg, and 20 μg of intrathecal fentanyl, respectively, and 1 patient received a dose of 100 μg in error. Demographic and clinical data for patients who did and did not receive intrathecal fentanyl are included in the Table. Nine patients had conversion to general anesthesia prior to skin incision, and 2 patients had conversion to general anesthesia after delivery of the baby. Of the 11 patients who required general anesthesia, 1 had a failed block with a documented T10 dermatomal level, 9 had a failed block without documentation of dermatomal level, and 1 patient did not have a reason documented for conversion to general anesthesia.

**Table.** Demographic and Clinical Data by Group

Variable	Received Intrathecal Fentanyl, n=161	Did Not Receive Intrathecal Fentanyl, n=600	*P* Value
Age, years	31 (26-34)	30 (26-34)	0.387
Height, cm	165.1(160-167.6)	162.6 (157.5-167.6)	0.059
	n=111	n=452	
Weight, kg	94.3 (77.4-112.4)	88.9 (75.2-104.0)	0.031
	n=119	n=493	
Body mass index, kg/m^2^	35.0 (30.1-41.1)	33.3 (28.9-38.9)	0.078
	n=106	n=452	
Gravidity	3 (2-3)	3 (2-4)	0.517
Parity	1 (1-2)	1 (1-2)	0.690
Gestational age, weeks	38.7 (37.7-39.1)	38.9 (37.6-39.1)	0.945
History of cesarean delivery, n (%)	Yes: 114 (70.8)	Yes: 431 (71.8)	0.798
	No: 47 (29.2)	No: 169 (28.2)	
History of depression, n (%)	Yes: 17 (10.6)	Yes: 66 (11.0)	0.873
	No: 144 (89.4)	No: 534 (89.0)	
History of anxiety, n (%)	Yes: 16 (9.9)	Yes: 72 (12.0)	0.468
	No: 145 (90.1)	No: 528 (88.0)	
Indication for cesarean delivery, n (%)			0.520
Elective	114 (70.8)	417 (69.5)	
Urgent	42 (26.1)	172 (28.7)	
Fetal heart rate abnormalities	5 (3.1)	11 (1.8)	
Attending anesthesiologist of record, n (%)			<0.001^a^
A	4 (2.5)	113 (18.8)	
B	1 (0.6)	91 (15.2)	
C	55 (34.2)	38 (6.3)	
D	11 (6.8)	101 (16.8)	
E	14 (8.7)	100 (16.7)	
F	36 (22.4)	81 (13.5)	
G	39 (24.2)	66 (11.0)	
H	0	4 (0.7)	
I	1 (0.6)	5 (0.8)	
J	0	1 (0.2)	
Operator who performed neuraxial anesthetic, n (%)	MD: 77 (47.8)	MD: 232 (38.7)	0.036
	CRNA: 84 (52.2)	CRNA: 368 (61.3)	
Neuraxial kit used for spinal anesthesia, n (%)	SSS: 152 (94.4)	SSS: 568 (94.7)	0.898
	CSE: 9 (5.6)	CSE: 32 (5.3)	
Intrathecal bupivacaine dose, mg	12.0 (11.3-12.0)	12.0 (11.3-12.0)	0.430
Received intrathecal morphine, n (%)	155 (96.3)	591 (98.5)	0.071
Intrathecal morphine dose, mg	0.2 (0.2-0.2)	0.2 (0.2-0.2)	<0.001
Time from spinal to surgery end, min	45 (39-50)	43 (38-49)	0.055
Conversion to general anesthesia, n (%)	2 (1.2)	9 (1.5)	1
Conversion to general anesthesia or anesthetic adjunct administration, n (%)	13 (8.1)	138 (23.0)	<0.001

^a^Monte Carlo analysis performed to determine Fisher exact test.

Notes: Data are presented as medians (interquartile ranges) unless otherwise noted.

CRNA, certified registered nurse anesthetist; CSE, combined spinal epidural; MD, physician anesthesiologist; SSS, single shot spinal.

Multivariate analysis designed to predict the odds of either conversion to general anesthesia or administration of systemic anesthetic adjunct medication between patients who did and did not receive intrathecal fentanyl is presented in the Figure. One hundred fifty-one patients either had conversion to general anesthesia or administration of systemic adjunct medication, and 610 patients did not. Fifty-eight physician anesthesiologists and 93 certified registered nurse anesthetists were the documented proceduralists who performed the spinal anesthesia for patients who either had conversion to general anesthesia or administration of systemic anesthetic adjunct medication compared to 251 physician anesthesiologists and 359 certified registered nurse anesthetists who were the documented proceduralists for patients who did not have either conversion to general anesthesia or administration of systemic anesthetic adjunct medication, a finding that was not statistically significant (*P*=0.540). Attending anesthesiologists C, F, and G used intrathecal fentanyl 59.1%, 30.8%, and 37.1% of the time, respectively, and collectively had conversion to general anesthesia or administration of systemic adjuncts at a rate of 14.0% compared to a rate of 22.0% for their colleagues, a difference that was statistically significant (*P*=0.004).

**Figure.**
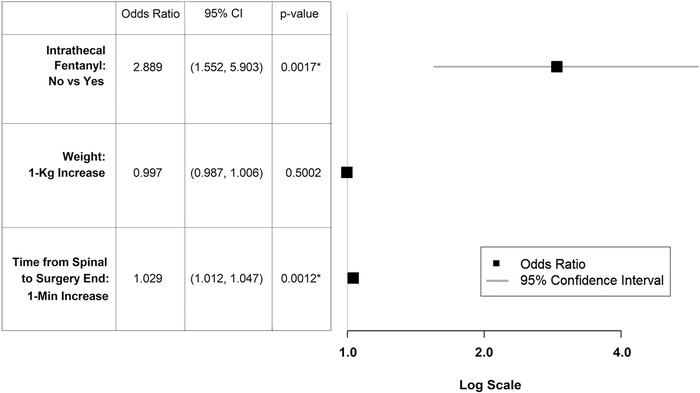
Multivariate model and forest plot show that subjects who did not receive intrathecal fentanyl had an approximate 189% increase in odds of either conversion to general anesthesia or administration of systemic anesthetic adjuncts. The area under the receiver operating characteristic curve for this model was 0.645. Asterisks denote significance.

## DISCUSSION

We found that only 21.2% of patients who underwent cesarean delivery with spinal anesthesia as the initial anesthetic technique received intrathecal fentanyl, and we attributed this finding to variances in the individual practice of the attending anesthesiologists of record. Only 10 attending anesthesiologists were included in our study, and we did not report any demographic data such as years of experience that could compromise their anonymity. We are unaware of any cost containment issues associated with restricting the use of intrathecal fentanyl for cesarean delivery. Our multivariate model showed that patients who did not receive intrathecal fentanyl had an approximate 189% increase in odds of either conversion to general anesthesia or administration of systemic anesthetic adjuncts.

The addition of intrathecal fentanyl to a spinal anesthetic has been shown to lower the dose of subarachnoid hyperbaric bupivacaine needed to provide adequate anesthesia for cesarean delivery. Choi et al demonstrated that patients who received 8 mg, 10 mg, and 12 mg of intrathecal bupivacaine reported intraoperative pain at rates of 35%, 20%, and 0%, respectively, while patients who received the same doses of intrathecal bupivacaine with the addition of intrathecal fentanyl 10 μg reported no intraoperative pain.^2^ A 2020 meta-analysis that included 14 randomized controlled trials with 694 patients undergoing cesarean delivery with spinal anesthesia showed that 17 of 370 patients (4.6%) who received intrathecal fentanyl with bupivacaine required systemic anesthetic adjunct medication compared to 96 of 324 (29.6%) patients who received only bupivacaine, a result that was statistically significant (*P*<0.001).^5^

Increased pain is a potential consequence of using lower doses of intrathecal bupivacaine for cesarean deliveries performed with spinal anesthesia. A 2011 meta-analysis showed a higher risk for systemic anesthetic adjunct supplementation in patients who received <8 mg of subarachnoid bupivacaine for cesarean delivery with spinal anesthesia compared to patients who received >8 mg.^6^ Studies published in 2004 and 2017 showed the ED95 (effective) dose of hyperbaric bupivacaine for cesarean delivery to be 11.2 mg and 12.6 mg, respectively.^7,8^ In our study, patients who did and did not receive intrathecal fentanyl had identical median doses of bupivacaine of 12.0 mg.

The effect of height and weight on the dose of intrathecal bupivacaine for cesarean delivery is controversial. Hogan and colleagues demonstrated that patients with increased abdominal pressure had lower volumes of cerebrospinal fluid,^9^ and this finding suggests that lower doses of intrathecal bupivacaine would be needed to provide spinal anesthesia for obese patients. However, Carvalho and colleagues found that patients with a body mass index >40 kg/m^2^ required a similar dose of intrathecal bupivacaine for cesarean delivery compared to nonobese patients.^10^ In our study, patients who received intrathecal fentanyl had a higher weight compared to patients who did not receive intrathecal fentanyl, but weight was not a predictor of either conversion to general anesthesia or administration of systemic anesthetic adjunct administration in our multivariate model.

The relationship between previous cesarean delivery and intraoperative cesarean delivery pain is unclear. Studies have demonstrated that patients with a history of cesarean delivery tend to have more adhesions and a longer operative time,^11,12^ but a study from 2019 showed that patients undergoing primary cesarean delivery had increased odds of incisional pain compared to patients undergoing repeat cesarean delivery.^13^ In our study, we found no difference in history of cesarean delivery between patients who received intrathecal fentanyl and those who did not.

Our study has several limitations. Height and weight data for patients were incomplete. The dermatomal level from spinal anesthesia was not routinely documented. We found that patients who received intrathecal fentanyl had a higher weight compared to patients who did not receive intrathecal fentanyl, and we used this variable in our multivariate analysis. The time from spinal placement to procedure end was close to meeting statistical significance, and we included this variable in our multivariate model. We found that patients who did not receive intrathecal fentanyl had a statistically significant higher percentage of certified registered nurse anesthetists who performed the neuraxial procedure compared to patients who received intrathecal fentanyl. We examined patients who received either conversion to general anesthesia or administration of systemic anesthetic adjunct medication and found no difference in the percentage of physician anesthesiologists or certified registered nurse anesthetists performing the neuraxial procedure. Therefore, we attribute our finding that nurse anesthetists were statistically more likely to be the operator for a spinal anesthetic that did not use intrathecal fentanyl vs a spinal anesthetic using intrathecal fentanyl to type I error.

## CONCLUSION

Intrathecal fentanyl should be strongly considered for use in spinal anesthesia for cesarean delivery in a community setting because of its ability to lower the risk of systemic anesthetic adjunct medication administration and conversion to general anesthesia.

## Notes

*This article meets the Accreditation Council for Graduate Medical Education and the American Board of Medical Specialties Maintenance of Certification competencies for Patient Care, Medical Knowledge, and Practice-Based Learning and Improvement.*
